# Treatment Strategies for Patients with Regional Odontodysplasia: A Presentation of Seven New Cases and a Review of the Literature

**DOI:** 10.3290/j.ohpd.a45070

**Published:** 2020-09-04

**Authors:** Pernille Hess, Eva Fejerskov Lauridsen, Jette Daugaard-Jensen, Nils Worsaae, Thomas Kofod, Nuno Vibe Hermann

**Affiliations:** a Senior Clinical Instructor, Department of Paediatric Dentistry and Clinical Genetics, School of Dentistry, Faculty of Health Sciences, University of Copenhagen, Denmark.; b Head Consultant, Resource Centre for Rare Oral Diseases, Copenhagen University Hospital, Rigshospitalet, Copenhagen, Denmark.; c Head Consultant Emeritus, Resource Centre for Rare Oral Diseases, Copenhagen University Hospital, Rigshospitalet, Copenhagen, Denmark.; d Specialist Consultant, Department of Oral and Maxillofacial Surgery, University Hospital, Rigshospitalet, Copenhagen, and the Regional Dental Care Unit, Capital Region, Denmark.; e Head Consultant, Department of Oral and Maxillofacial Surgery, University Hospital, Rigshospitalet, Copenhagen, and the Regional Dental Care Unit, Capital Region, Denmark.; f Associate Professor, Department of Paediatric Dentistry and Clinical Genetics, School of Dentistry, Faculty of Health Sciences, University of Copenhagen, Denmark.

**Keywords:** case report, dental anomaly, regional odontodysplasia, treatment

## Abstract

**Purpose:**

Regional odontodysplasia (RO) is a rare dental anomaly affecting primary and/or permanent dentition, and leads to comprehensive treatment need. The purpose of this study was to present a larger consecutive sample with RO, discuss treatment strategies for patients with RO, and review the literature.

**Materials and Methods:**

A consecutive, retrospective sample of seven children with RO (6 males, 1 female) including all patients diagnosed with RO in the eastern part of Denmark was conducted over a period of 15 years. The evaluation included gender, localisation and treatment outcome. A review of the literature and cases published within the last 15 years was conducted.

**Result:**

Referral age was 2-12 years (mean: 7.3 years). The gender ratio was 1:6 (female:male), and the right:left ratio was 3:4. 71% of the patients had RO in the mandible and 29% in the maxilla. 43% had RO in the permanent dentition, while both primary and permanent dentition were affected in 57%. Typically, RO affected incisors and canines. In some patients, RO also affected more distal tooth types. Treatment included early multiple extractions and subsequent combined orthodontics, surgery and prosthetics. A search on RO cases published within the last 15 years was conducted and included 44 cases. The review showed a male and maxillary preponderance. The most common treatment of RO is extraction.

**Conclusion:**

Treatment of RO should take place in interdisciplinary, specialised teams, and individual treatment plans should be designed. Fewer but more extensive treatment sessions under general anesthaesia may minimise the burden of care for the patients.

Regional odontodysplasia (RO), also known as ‘ghost teeth’, is a rare dental anomaly. The first case was described by Hitchin in 1934.^25^ In 1970, Pindborg suggested using the term regional odontodysplasia (RO), as the condition often involves several teeth in one region.^41^ RO is the most commonly used term today.

Both primary and permanent dentition may be affected. If primary teeth are affected, permanent successors will most likely have RO. However, affected permanent teeth do not necessarily have affected predecessors.^2,14,34,35^ Most often, a single region is affected, but the condition may cross the midline. Where this is the case, the condition commonly includes the contralateral central incisor. Few cases involve only a single tooth. RO is typically diagnosed at the age of three to four years (time of completed eruption of primary dentition) or around ten years of age (time of eruption of the permanent incisors and canines).^2,20,33,34,35^

Typical clinical signs of RO are failure of eruption, discoloured and soft teeth with irregular shape, plus surface pits and grooves often accompanied by gingival swelling and tooth abscesses. Accordingly, patients usually present with pain.^14,20,33^ Both the enamel and the dentin are affected, as the anomaly affects the mesodermal and ectodermal elements of the dental organ.^8^

Radiographically, teeth present a shadow-like structure with reduced radio-density, giving the anomaly the nickname ‘ghost teeth’. There is no clear distinction between hypomineralised enamel and hypomineralised dentin. Roots are short, and both pulp chambers and the apical foramen appear wide, which makes them look immature.^1,2,20,33,49^

Like the distinctive radiological signs, the histological findings in RO are characteristic: All dental germ structures are affected, although the coronal elements are more affected than the radicular ones. A typical finding is hypoplastic, hypomineralised and matrix-enriched enamel of variable thickness, generally thin with irregular enamel prisms. The dentin layer is also reduced in thickness, consisting of areas with globular and irregular interglobular spaces, as well as cellular and amorphous areas. Coronal dentin is fibrous, consisting of clefts and fewer dentinal tubules; radicular dentin is generally more normal in terms of structure and mineralisation. The tooth follicle is thickened and fibrous, showing swirled cell disturbances, including small islands of odontogenic epithelium and spherical calcifications. The pulp often contains denticles and amorphous mineralised material.^14,19,20,24,33,35^

The aetiology of RO remains unknown. Causal factors may include trauma, local circulatory disorder, infection, viral infection, neural disturbance, metabolic and nutritional disturbance, or vitamin deficiency, but they have all been precluded.^10,14,20,24,34^ Currently, no single triggering factor is acknowledged. Thus, the occurrence of RO must be considered idiopathic. The literature suggests no hereditary origin, and RO is not related to ethnicity. The gender ratio shows a slight female preponderance with a female to male ratio varying from 1.4:1^34^ to 1.7:1^49^. The condition is rare and no precise prevalence has been determined, since existing studies are based on case reports.

The overall aim of the present retrospective study was to present a consecutive sample of patients with RO and discuss different treatment strategies. Furthermore, to evaluate the occurrence of RO according to gender, location and treatment, an overview of published RO-cases during the last 15 years is presented.

## Materials and Methods

### Sample

The retrospective, consecutive mixed-longitudinal sample included seven patients with RO seen within the last 15 years at the Resource Centre for Rare Oral Diseases (RCROD), Copenhagen University Hospital, Rigshospitalet, Copenhagen, Denmark. RCROD receives referrals from municipal dental care and private dentists in the eastern part of Denmark. The team is multidisciplinary, consisting of paediatric dentists, prosthodontists, orthodontists, and oral surgeons.

### Methods

Due to the small sample size, this study is purely descriptive.

In this study, the RO diagnosis was based on the clinical and radiological findings. Gender, location affected, and treatment strategies were then registered, evaluated, and compared. In all patients, a full medical history, clinical and radiographic examination (including panoramic and periapical radiographs), as well as clinical photos were obtained at the first visit at RCROD. Subsequent radiographs and clinical photos were taken as individually indicated in connection with control or treatment. If present, previous patient records and radiographs were acquired from the referring dentist.

### Medical History and Clinical Examination

A full medical history was obtained, including information about previous serious diseases/illnesses, use of medicine, allergies, familial dental abnormalities, etc. The clinical examination included chronological and dental age, dental status, and evaluation of tooth morphology, shape and structure of erupted teeth, as well as gingival findings and symptoms from teeth, such as pain.

Clinical criteria for diagnosis of RO were: delayed (more than 6 months) or non-eruption of teeth in a localised region. Erupted teeth showed hypomineralised enamel, discoloured enamel (yellow-brown), and soft enamel structure with irregular shape plus surface pits and grooves. Gingivae exhibited swelling.

### Radiography

A panoramic radiograph was used for evaluating the region and extent of the condition, number of affected teeth, and whether or not permanent successors were affected. Single tooth projection was used for collection of more details on enamel and dentin structure and their relation.

The radiographic criteria for diagnosis of RO were: no clear distinction between hypomineralised enamel and hypomineralised dentin, a shadow-like structure with reduced radiodensity, and short roots with both a wide pulp chamber and apical foramen.

### Histology

In two patients (D and G), a histological examination of extracted teeth was performed. The histological analysis included description of the degree of mineralisation of enamel and dentin as well as surrounding tissue.

### Ethical Considerations

Informed consent was obtained from all children’s parents/guardians. All data used in the present study were obtained in a clinical context as part of a standardised treatment protocol with full acceptance from the parents, and in accordance with the World Medical Association Declaration of Helsinki, 2013.

By Danish law, this study is a ‘quality assurance study’ (all data were obtained in a clinical context and/or as part of a standardised treatment protocol). Hence, the study did not qualify for evaluation in a research ethics committee in Denmark.

## Results

### Sample

A total of 7 patients (1 female and 6 males) was included in the study, thus the gender ratio was 1:6 (female:male). Age of referral was between 2 and 12 years (mean 7.3 years). The age of diagnosis was between 2 and 12 years (mean 5.6 years). The follow-up time ranged from 0 to 19 years (mean 8.4 years). Three of the patients completed their treatment in their early twenties. A short description of all patients and their treatment is given in Table 1.

**Table 1:** Overview of the sample, including RO affected teeth, treatment and follow-up time

Patient	Age in years at diagnosis (D) referral (R) l ast evaluation (E)	Period of observation (years)	Sex	Jaw	Teeth affected	Treatment	Comments/other observations
A	D: 2 R: 2 E: 2	0 years	Male	Maxilla, right	51, 52, 53, 54, 55 11,12, 13, 16	Extraction due to fistulas 51, 54, 55	Severe lung infection + several other infections in early childhood
B	D: 2.5 R: 2.5 E: 2.5	0	Male	Mandible, left	71, 72, 73 31, 32, 33	Extraction planned due to abscess 73 Extraction planned to prevent future abscess/pain 71, 72	Patient moved away; no follow-up.
C Fig 1	D:4.5 R: 4.5 E: 10	4.5–10 years = 6 years	Male	Maxilla, left	61, 62, 63, 64, 65 21, 22, 23, 24, 25, 26	Extraction 61, 62, 63, 64, 65: cystic tissue surgically removed Surgically removed/extraction 21, 22, 23, 24, 25, 26: 3 implants inserted at age 6 Orthodontic treatment + onlays on implants at age 9	85: Extracted due to pain, crumbling. Cyst 46
D Fig 2	D: 8 R: 12 E: 18	8–18 years = 10 years	Female	Mandible, right	41, 42, 44, 46	Extraction 46 Endodontic treatment 41, 42 failed, hence extraction Extraction 83 1 implant at age 17 Interpositional sandwich osteotomy Orthodontic treatment, with implant anchorage 2 implants, 1 crown and a 3-unit bridge at age 18	Histological examination of extracted teeth Agenesis 45
E	D:7 R: 10 E: 21	10–21 years = 11 years	Male	Mandible, left	31, 32, 33,	Extraction 31, 32, 33 Orthodontic treatment Bone augmentation 2 implants + crowns at age 21	
F Fig 3	D:12 R: 12 E: 24	7–24 years = 13years.	Male	Mandible, left	31, 32, 33	Extraction 31, 32, 33 Removal of tumour tissue/cyst Extraction 73 due to treatment 33 Orthodontic treatment Bone augmentation 2 implants + 3-unit bridge at age 24	Several ear infections, grommet. Father has agenesis permanent maxillary lateral incisors
G Fig 4	D:3 R: 8 E: 21	3–21 years =19 year	Male	Mandible, right	81, 82, 83, 84, 85 41, 42, 43, 44, 45, 46, 47	Extraction 81, 84, 85 Extraction of teeth/tooth germs 41, 42, 43, 44, 45, 46, 47 Autotransplantation 15, 25 to regions “45”, “44” at age 13 Extraction 82 Autotransplant in region “44” later lost due to ankylosis Orthodontic treatment with implant anchorage Bone augmentation Patient is currently awaiting orthognathic surgery, implants and final restoration at age 21	61, 62: Surgical removal to promote eruption 21, 22: Denudation to promote eruption 53, 54, 63, 64: Extracted for orthodontic reasons Histological examination of extracted teeth Behavioral management problems, treatment fatigue

### Medical History

With the exception of having RO, all patients were healthy individuals. None of the patients showed signs of caries activity in either the primary or permanent dentition, and in the total sample only a single restoration of a first permanent molar was observed, and this tooth was not affected by RO (patient C).

However, it should be mentioned that one patient had suffered from a severe lung infection as a newborn and several infections in early childhood. One patient had suffered from severe otitis media and had frequently received antibiotics; subsequently, a grommet was inserted and the tonsils were removed. One patient had asthmatic bronchitis as a toddler.

In general, there was no family history of dental abnormalities in the sample. However, in a single case, the father showed agenesis of both maxillary lateral incisors.

### Clinical and Radiological Findings of RO

#### Jaws affected and right-/left-sidedness

All patients had either the right or left jaw affected. In no case did the condition cross the midline.

Three of the patients had right-sided RO and four had left-sided RO, i.e. a right:left ratio of 3:4. Five of seven patients had RO in the mandible (71%) and two in the maxilla (29%), i.e. the condition was 2.5 times more common in the mandible than in the maxilla. None of the patients had RO of both the mandible and maxilla.

#### Affected dentition

In three of the patients, only the permanent dentition was affected (43%), and in 4 patients, both the primary and permanent dentitions were affected (57%). Patient records and radiographs were acquired from the general dentist to confirm that primary teeth had not been affected. When both dentitions were affected, the referral age was 4 years, but 11 years of age when only the permanent dentition was affected, which is a common finding.^2,20,33,34,35^

#### Affected tooth types

In all patients in the sample, RO affected both permanent central and lateral incisors. This finding was not associated with the affected dentition (permanent only vs primary and permanent dentition) or jaw. In some patients, the first and second premolars were also affected (N=3), as well as the first and second permanent molar (N=4). In two patients. RO also affected the first and second primary molar. See Table 1 for an overview of RO-affected teeth.

### Histological Findings

All histologically examined teeth showed hypoplastic, hypomineralised, and matrix-enriched enamel of variable thickness and irregular construction. The enamel-dentin border was scalloped, and in the dentin, a variable pattern, size and number of dentinal tubuli were seen, with large amounts of interglobular dentin in an irregular pattern.

The periodontal membrane and the tooth follicle were quite cellular with single focal, whirled nodules and a number of islands of odontogenic epithelium. On the surface of the enamel or in the pulp tissue, calcified structures were present. The connective tissue was quite cellular and had a nodular structure. These findings confirmed the RO diagnosis.

### Treatment

**Fig 1:**
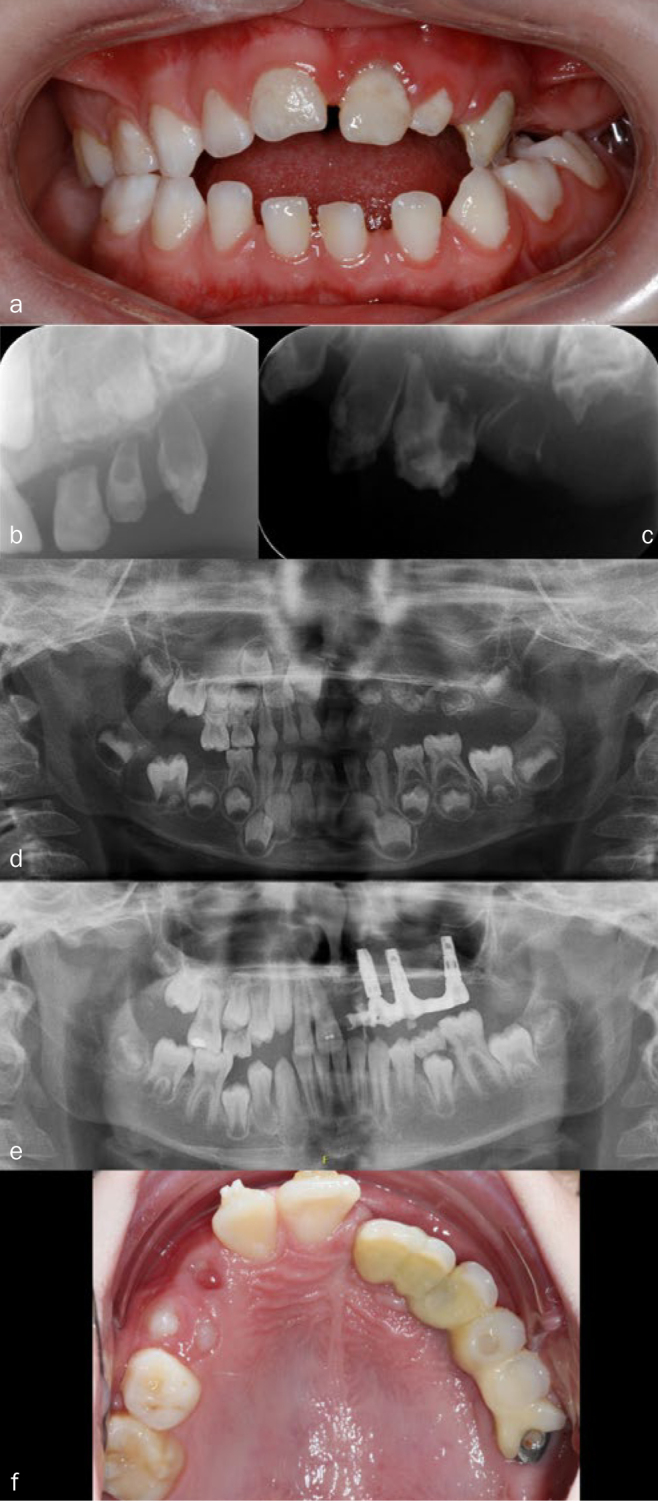
Treatment of patient C, followed-up for 6 years (from 4.5 to 10 years of age). a) Clinical photo at age 4.5 years. b) + c) Radiograph at age 4. d) Orthopantomogram at age 4.5. RO was seen on the maxillary left side affecting 61, 62, 63, 64, 65, 21, 22, 23, 24, 25, and 26. In addition, a cyst was seen in region 46. 75 initially in supraposition. e) Radiograph taken at age 9 (3 years after surgery) shows three implants in the RO affected region. Cyst gone and 46 erupted normally. f) Clinical photo of teeth at age 9. Note the shift of the midline.

**Fig 2:**
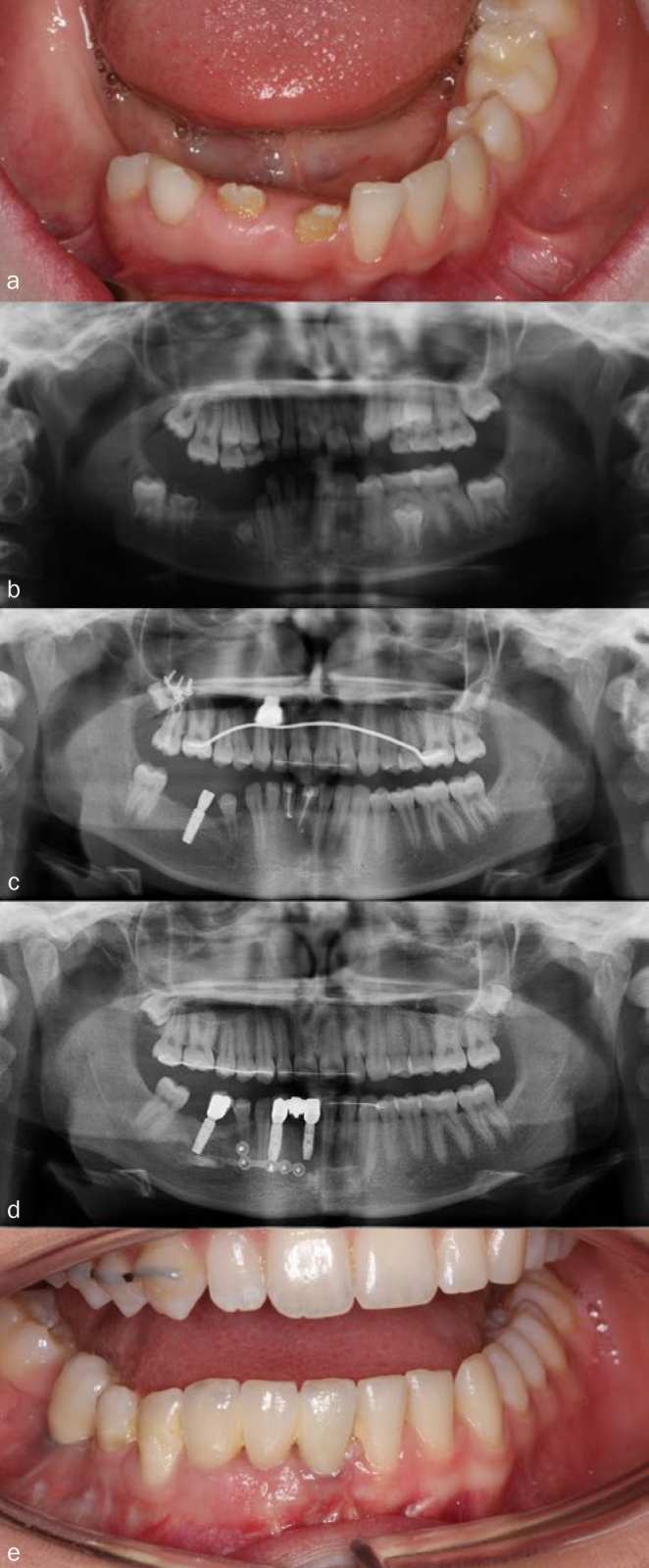
Treatment of patient D, followed for 10 years (from 8 to 18 years of age). a) Clinical photo at age 12. Teeth 42 and 41 were yellow, pitted and irregular shaped, 83 and 43 are present. b) Orthopantomogram at age 11. Radiologically, teeth 46, 44, 42 and 41 have an unusual shape, small in size with large pulp chambers. In addition, agenesis of 45. Note supraposition of teeth in 1st maxillary quadrant. c) Orthopantomogram at age 14. Teeth 44 and 43 have erupted between affected teeth in the region. An implant was inserted region 45. Endodontic treatment of 41 and 42. d) Orthopantomogram at age 18. 41, 42, 83 extracted. Two implants, inter positional sandwich osteotomy, to level the right side of the mandibular occlusal plane. e) Clinical photo of the teeth with crown and the 3-unit bridge in place.

**Fig 3:**
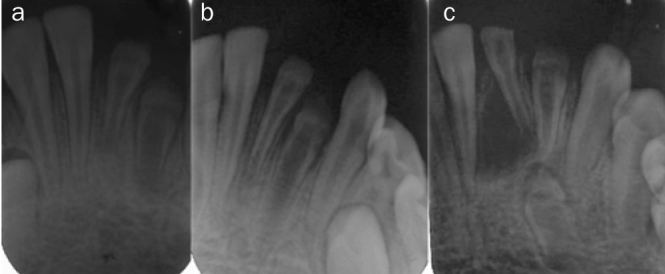
Treatment of patient F, followed for 13 years (from 7 to 24 years of age). Teeth 31, 32, and 33 are affected by RO. a) to c) Radiographs taken at age 9, 10 and 12 visualise maturation and a slow eruption of 31, 32 and 33 and development of a radicular cyst.

**Fig 4:**
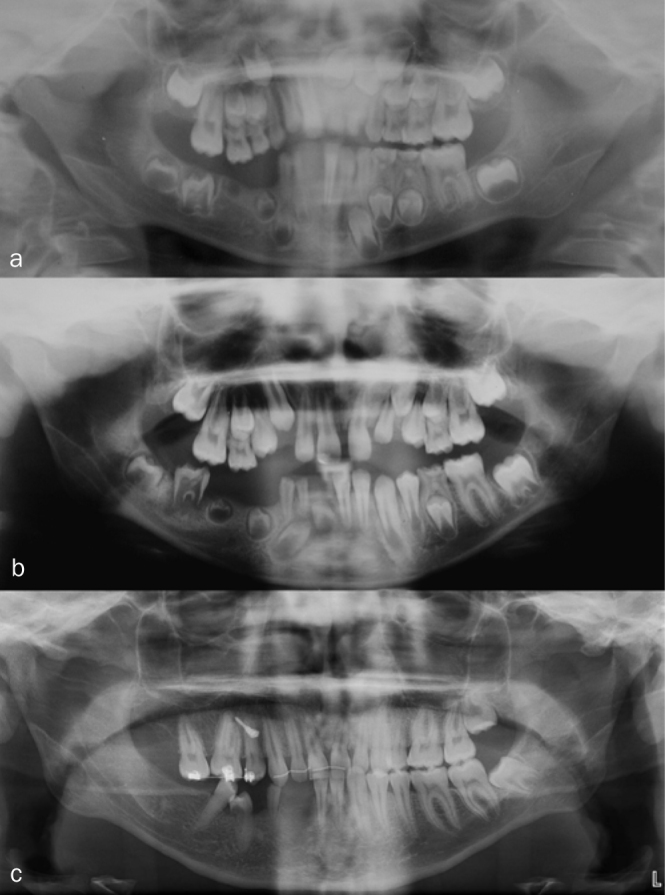
Treatment of patient G, who was followed for 19 years (from 3 to 21 years of age). a) Orthopantomogram at age 8. Teeth 47, 46, 45, 44, 43, 42, and 41 are affected by RO. Note the supraposition of teeth in the maxillary right quadrant. b) Orthopantomogram at age 10. Tooth 46 is erupting despite minimal root formation. c) Orthopantomogram at age15. Teeth 47, 46, 45, 44, 43 and 42 extracted. Autotransplantation of 15 and 25 to region 45 and 44. Note the radiolucency on ‘44’, this tooth was latter lost due to ankylosis.

The treatment plan/regime for all patients included multiple extraction and/or surgical removal of primary and/or permanent teeth due to RO alone. Extraction/surgical removal of the permanent teeth was typically followed by later insertion of implants with or without bone augmentation and orthodontic treatment. It should be noted that two of the patients were only about two years old, so that future treatment needs involving the permanent dentition are difficult to foresee. Figures 1-4 show examples of some of patients with long-term follow-up.

#### Extraction and surgical removal

In the current sample, we found that in patients with primary dentition affected by RO, a total of 16 teeth were extracted due to RO alone. The mean extraction number for each patient was 4 primary teeth.

For patients in whom RO affected the permanent dentition, a total of 22 teeth were extracted due to RO alone, with a mean extraction number for each patient of 4.4 permanent teeth. Abscesses provided the indication for the first extraction, but often several teeth were extracted concurrently to minimise the number of appointments.

#### Implants

Five patients in whom RO affected the permanent dentition were treated with at least 2 implants in their teens, early twenties or before, with a mean age 18. One patient had implants inserted at 6 years of age. Excluding this patient, the mean age for implant insertion is 20. Three patients underwent bone argumentation prior to insertion of implants.

#### Auto-transplantation

One patient had autotransplantation at age 13. Teeth 15 and 25 were transplanted to region 45, 44. Orthodontic treatment followed. Three years later, tooth 44 was extracted due to ankyloses and resorption.

#### Orthodontic treatment

Five patients in whom RO affected the permanent dentition underwent orthodontic treatment in their teens, and one patient had orthognathic surgery.

### Previous Literature

**Fig 5:**
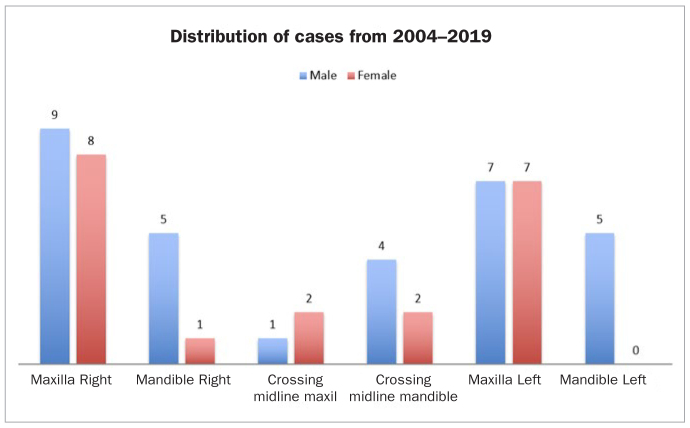
Distribution of gender, jaw and side in patients with RO in published cases from Tables 1 and 2.

To determine the number of published cases, a literature search was performed in PubMed with the search word ‘regional odontodysplasia’. Published cases from April 2019 back to January 2003 were included. This year was chosen because Tervonen et al^49^ conducted a review including cases published up until 2002. This generated 120 hits. Articles were sorted using the inclusion criteria: headline containing RO and articles comprising at least one case report. Only English articles and only those case reports with radiographs supporting the diagnosis were included. The search based on these criteria yielded 39 articles including 44 cases. These articles and cases are listed in Table 2. Figure 5 graphically depicts the distribution of gender and location of the cases listed in Tables 1 and Table 2.

**Table 2:** Data from published cases from April 2019 back to January 2003

Author	Year/country	Age at diagnosis/gender	Location and affected teeth	Treatment	Other findings
Silva Cunha et al	2019 Brazil	10/Female	Maxilla Right side 53 17, 16, 15, 14, 13	Extraction 53, 17, 16, 15, 14, 13 Functional rehabilitation Implants and prosthetic rehabilitation planned when growth complete	Histological examination
Koskinen et al	2019 Finland	6/Female	Maxilla Right side 55, 54, 53,52 16, 13, 12, 11	Not described in detail Removable denture	Screening of genes, PAX9 Agenesis 17,15,14, 25, 27 Mother and sister multiple agenesis
Koruyucu et al	2018 Turkey	6/Female	Maxilla Left side 21, 22, 23	Temporary prosthetic rehabilitation Periodontal surgery Orthodontic treatment Endodontics, MTA. Fiber posts Zirconia crown	
De Sá Cavalcante et al	2018 Brazil	8/Male	Mandible Right side 81,82,83,84,85 43, 44, 45, 46	No follow-up	Agenesis 41,42 Caries 83, 84, 85, 46 CBCT
Bowden et al	2018 United Kingdom	3/Female	Maxilla Left side 61, 62, 63, 64, 65, 26	Extraction 61, 62, 63, 64	
Al-Mullahi and Toumba	2016 United Kingdom	5/Female	Maxilla Right side 55, 54 15, 16, 17	Extraction 55 Stainless steel crown 54, 64 Later on extraction 54 and root remnants 55	Generalised enamel defects 64, 74, 72 Histological examination
Jahanimoghadam et al	2015 Iran	5/Female	Maxilla Right side 55, 54, 53, 52, 51, 17, 16, 15, 14, 13, 12, 11	Age 5-6: Extraction 55, 54, 53, 52, 51 Acrylic appliance At age 10: Extraction 12	Blood count (all normal) Histological examination 12
Mathew et al	2015 India	10/Female	Maxilla Left side 26, 25 65	Extraction 26 due to infection Observation (25 not yet erupted)	65 lost 2 years before referral. Histological examination 26
Babu et al	2015 India	33 month/Male	Mandible Right and left side 71,72,73,74,75,36 81,82,83,84,85,46	Observation	Blood count (all normal)
Matsuyama J. et al	2014 Japan	5.8/Male 6.1/Male	Maxilla Left side 61, 62, 63, 65, 21, 22, 23, 25 Maxilla Right side 51,52 11,12	51, 52, 53 extracted prior to examination Removable space retainer Age 9 computed tomography (CT) 51,52 did not exfoliate like 11, 12 erupted hence extraction 51,52 was done. Age 9 CT	CT assessment of the enamel, dentin and follicle values 54, 24 not affected 26 hypoplastic CT assessment of the enamel, dentin and follicles values
Rashidian et al	2013 Iran	3.5/Female	Maxilla Left side 61, 62, 63, 64, 65 26	Suggested treatment plan: Extraction 64 Stainless steel crown 65 (with crown, band and loop) Glasionomer 61,62,63	
Al-Tuwirqi et al	2014 Australia	7/Male	Mandible Right side 85, 43,44, 46,47	Surgical removal 46 and cyst	Cyst-like radiolucency 46 Extraction 16 due to overeruption
Erpardo et al	2012 USA	12/Female	Maxilla Left side 61, 21, 23, 24, 25, 26	Surgical enucleation + gingivectomy	Normal 22 between RO affected teeth
Ziegler et al	2012 Germany	7.5/Male	Mandible Left side 71, 72, 73 31(mild), 32, 33	At 11.8 year extraction 32, 33, 64 and Autotransplantation 25 to region 33 1 year later: Extraction 31, autotransplantation 15 to region 31	Early loss 72, 73
Ganguly et al	2012 USA	18 /Male (but 14 years old at the time of first radiographs)	Mandible Right and left side 43, 33, 34, 35, 36,3 7 31, 32 are missing		Natural maturation and development was seen in the RO-affected teeth
Barbería et al	2012 Spain	7/Male 13/Male 4/Male (Russian)	Mandible Right side 81, 82, 83, 84, 85 41, 42, 43,46 maybe 44, 45. Mandible Left side 71, 72, 73, 74, 75 31, 32, 33, 34, 35, 36 Maxilla Right side 51, 52, 53, 54, 55 11, 12, 13, 14, 15, 16, 21	82, 83, 84 extracted due to infection at age 3.4. Partial denture mandibular right side. At age 3 71, 72, 73, 74, 75 were extracted. Partial denture mandibular left side Extraction 51, 52, 53, 54, 55 Partial denture maxillary right side	Perinatal encephalopathy and rachitism Frequent ear infections right side
Canela et al	2012 Paraguay	10 month/Male	Maxilla Left side 61, 62, 63, 64, 65 21, 22, 23, 24, 25, 26, 27	Control every 2nd month, followed up only 5 years 64 extracted due to abscess	
Gurunathan et al	2011 India	11.6/Male	Maxilla Right side 11	Extraction 11 Partially removable denture	Histological examination
Mehta et al	2011 India	12/Female	Maxilla Right side 11, 12, 13, 14, 15, 16, 17	Extraction 11, 12, 13, 14, 15 Partial acrylic denture	
Gallo et al	2011 Brazil	2.5 /Female	Maxilla Right side 51, 52, 53, 54, 55 11, 12, 13, 14, 15, 16	Conservative plan initially, but 2 months later 54 and 51 were extracted due to pain. Later extraction of both primary teeth 52, 53, 55 and permanent dental germs 11, 12, 13, 14, 15, 16. Removable appliance	Histological examination
Upadhyay et al	2011 India	13/Female	Maxilla Right side 11, 12, 13, 15, 16, Less affected 14, 21		
Thimma Reddy et al	2010 India	5/Male	Maxilla Right side 51, 52, 53, 54, 55 11, 12, 13, 14, 15, 16	Extraction 51, 52, 53, 54, 55 Acrylic removable appliance Periodical follow-up	Caries 64,65, 71,74,75,84,85
Pugalagiri and Kessler	2010 USA	4/?	Maxilla Right side 55, 14, 15, 16		
Quinderé et al	2010 Brazil	8/Male	Maxilla left and Mandible right and left side 17, 16, 15, 14 47, 46, 45, 44, 43, 42, 41, 31, 32, 33	Endodontic treatment 16, 26, 36, 46, 31, 41	Vascular nevus
Gondim et al	2009 Brazil	1.5/Male	Maxilla Right side 51 ,52, 53 11, 12	Extraction due to abscess 51, 52. 53 fluoride, monthly control Removable acrylic appliance	Histological examination
Ferguson et al	2009 USA	20 months / Female	Maxilla Left side 61 ,62 ,63 21, 22, 23	Abscess age 3.5 led to antibiotics and extraction 61, 62, 63 7-14 years: different orthodontic treatments, with acrylic replacement 21, 22 18 years: extraction 21, 22, 23, Bio-Oss+Bio-Guide 19 years: Autogenous corticocancellouos graft. 20 years: 2 implants, 3 porcelain veneers	Dysplasia mesial 11
Kappadi et al	2009 India	14/Female	Maxilla Right side 11, 12, 13	Extraction 11, 12, 13 Temporary acrylic partial denture	Trauma age 6 with avulsion of a few deciduous teeth and few fractured teeth which were removed. 21 and 16 are missing possibly due to this Histological examination
Magalhães et al	2007 Brazil	5/Female	Maxilla Left side and 11 61, 62, 63, 64, 65, 51 11, 21, 22, 23, 24, 25, 26, 27	61, 64, 65, 51 extracted and 63 filled prior to referral. Extraction 62 Partial acrylic denture	Mother took Enapril Haemangioma right side 36 developmental anomaly
Gündüz et al	2008 Turkey	8/Male	Maxilla Right side 51, 52, 53 11, 12, 13	Extraction 11, 53 Temporary acrylic denture	Histological examination Caries in the other primary molars
Carlos et al	2008 Guatemala	12/Female 25/Male	Maxilla Right and left side 11, 12, 13, 14, 15, 16, 21, 22 Mandible Right and left side 43, 42, 41, 31, 32, 33, 34, 35, 36 (Mother reported affected primary teeth as well)	Surgical removal 11, 12, 13, 14, 15, 16, 21, 22 Prosthetic rehabilitation Surgical removal 43, 42, 41, 31, 32, 33, 34, 35, 36 Prosthetic rehabilitation	Histological examination Histological examination Endodontic treatment 31,32 – though not described in the article
Volpato et al	2008 Brazil	12/Female	Mandible Right and left side 47, 46, 45, 44, 43, 42, 41, 31, 32, 33	Extraction Provisory prosthetic rehabilitation	Histological examination confirming the diagnosis Red mark right side of the face at birth vanished after 1 month; mother fell during pregnancy
Spini et al	2007 Brazil	7/Male	Mandible Right side 45, 44, 43, 41 42 just a small affection Primary dentition not described	Left affected teeth in the bone for 7 years to promote bone growth. Extraction due to abscess 41, 42, 83 45 erupted 43, 44 extracted. Prosthetic rehabilitation	Sister had a maxillary osteoma Histological examination of the affected gingiva revealed a haematoma
Cho et al	2006 China	10/Male	Maxilla Right side 11, 14, 15, 16 13 (to a lesser extent)	Extraction 16 Composite resin 11, 13 Observation	Continuing root formation in teeth with RO RO skippped a tooth in row of affected teeth
Rosa et al	2006 Brazil	8/Male	Maxilla Left side 61, 63 21, 22, 23	Extraction 61, 63 Filling 65, 26 1 year later extraction 21, 22, 23 Prosthetic rehabilitation	65 caries lingual 26 hypoplastic Fall history age 2 Biopsy and histological examination
Cahuana et al	2005 Spain	5/Male 3/Female	Maxilla Right side 55, 54, 53 11, 12, 13, 14, 15 Maxilla Left side 64,65,24,25,26,27	Scaling 2 month later pain which led to extraction 55, 54, 53 Removable acrylic appliance At age 10 extraction 14, 15, 16 and autotransplantation 24, 34, 44 Observation (now age 7)	52, 51 fracture due to trauma 61 avulsion
Özer et al	2004 Turkey	5/Male	Maxilla Left side 61, 62, 63, 64, 65 11, 21, 22, 23, 24, 25, 26, 27	Extraction 65, 61, 62 resulting in an edentulous quadrant Removable acrylic appliance	
Hamdan et al	2004 Jordan	8.5/Female	Mandible Right and left side 81, 82, 83, 71, 72, 73 41, 42, 43, 31, 32, 33	Surgical removal 81, 82, 71, 72 Temporary acrylic partial denture	Histological examination
Tervonen et al	2004	The affected teeth are not listed here, as they are already included in the ratio			
Chinn et al	2003 Columbia	2/Female	Maxilla Left side 61,62,63	Extraction 61,62,63 in general anaesthesia and sealants 64, 74, 84	Histological examination
Courson et al	2003 France	11/Male	Maxilla left and right side 13, 12, 11, 21		Histological examination

## Discussion

The following discussion is based on the presented sample. Although it is the largest consecutive sample in the literature, it is still a small, which limits the strength of the results.

In the present sample, which includes all seven patients diagnosed with RO within the past 15 years at the RCROD, the gender ratio was 1:6 (female: male) (Fig 5). This does not concur with the female preponderance reported in previous studies.^8,20^ Tervonen et al^49^ found a female:male gender ratio of 1.7:1, while Crawford and Aldred^14^ reported a female:male ratio of 1.4:1. The female:male ratio based on the cases from both Tables 1 and 2 is 1:1.7. This shows male preponderance, which does not agree with the previously reported gender distribution. Whether or not the shift from a female to male preponderance is due to coincidence or statistical uncertainty is not known. However, gender should not be considered crucial.

In contrast to previous reports in the literature showing maxillary predominance,^8,16,20^ mandibles were predominant in our sample. Tervonen et al^49^ found the maxilla:mandible ratio to be 1.6:1. Lustman and Ulmansky^34^ found that the maxilla was affected twice as often as the mandible, and Crawford and Aldred^14^ found the maxilla:mandible ratio to be 2.5:1. As shown in the published cases in Table 2 and our cases in Table 1, the maxilla:mandible ratio was 1.6:1. The overall data support previous findings that the maxilla is affected more frequently. The reason for this is unknown.

RO generally affects one quadrant, but may cross the midline and may affect both the right and the left side.

There is no global overview of all diagnosed cases, nor is it known how many cases are undiagnosed. Consequently, the proposed jaw and gender distributions can be considered only estimates, as they are based on case reports only, and only those written in English. It is therefore unknown whether these distributions are representative for the population with RO.

When a quadrant is affected by RO, it usually affects consecutive teeth, although in rare cases normal teeth may erupt in an area with RO. In case D, a healthy tooth, 43, erupted between RO-affected teeth. Tooth 44 was only mildly affected with pitted, slightly discoloured enamel. Similarly, this rare phenomenon has been described by Al-Tuwirqi et al^3^ where 45 erupted, though delayed, compared with the other side. Cho^12^ reported a case where 12 erupted with no anomaly and 13 was only mildly affected, while 11, 14 and 15 were severely affected with RO.

The fact that in most cases RO affects all teeth in a developmentally associated region may indicate that the condition could be related to the specific tooth ontogeny/ innervation. However, the reported cases of normal, RO-unaffected teeth erupting between affected ones suggests that the condition in these cases may be explained by a local phenomenon of another origin. It is plausible that the phenotype of teeth with RO varies greatly, with some very mildly affected subtypes.

Genetics and epigenetics may hold the key to a better understanding of cause and correlation of RO. In this effort, analysis of histological sections and search for specific enzymes or regulatory factors in dental hard tissues and the surrounding soft tissue may be interesting.

Courson et al^13^ suggested their findings of an increased amount of matrix metalloproteinases as a possible origin of RO. Furthermore, based upon SEM and TEM, Carlos et al^10^ suggested that RO was characterised by interruption of normal ameloblastic function in a specific period of odontogenesis. Koskinen et al^31^ showed changes in PAX9 similar to patients with multiple agenesis or oligodontia.

In rare diseases like RO, it may be difficult to achieve evidenced-based treatment or, indeed, just to establish best practice. Currently, the treatment of RO is based on individual considerations. The literature consists mainly of case reports addressing mostly diagnostics and acute or initial treatment at very young ages; only a few case reports consider aspects of long-term treatment (Table 2). Different treatment approaches were used in our sample, based on the knowledge gained from the first to the latest referral. The team at RCROD is multidisciplinary, consisting of paediatric dentists, prosthodontists, orthodontists and oral surgeons who supplement each other in diagnostics and treatment.

**Fig 6:**
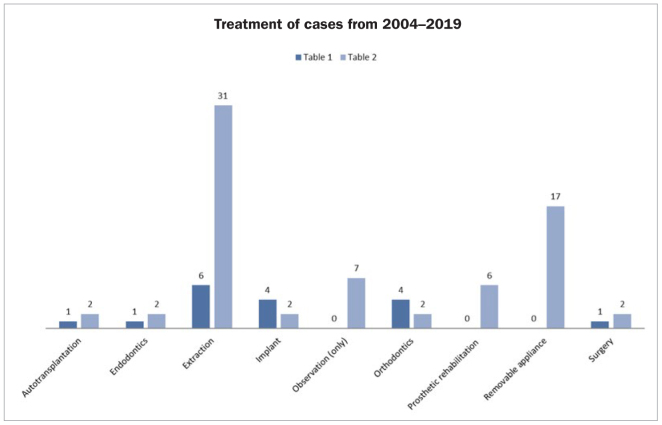
Distribution of treatment in patients with RO in published cases from Tables 1 and 2.

The treatments listed in Tables 1 and 2 are visualised in Fig 6. The most common treatment, 100% in Table 1 and 70% in Table 2, is extraction of RO-affected teeth, either all together or separately when presenting with pain or when prior treatment has failed. In our cases, RO-affected teeth were extracted separately in the earliest cases, but together in the latest ones (the accumulated experience indicated later extraction). The overall purpose of this treatment strategy was to minimise numbers of treatments and to avoid children presenting with pain due to abscess. A disadvantage of this regime may be that teeth which could potentially have been preserved and erupted are extracted.

In the literature, eruption was followed in seven cases.^4,9, 12,15,18,37,48^ In two cases, eruption succeeded.^12,18^ In another case, merely one tooth was extracted during a five-year period.^9^ The rest of the cases needed follow-up later on due to early age at diagnosis. A good argument for preserving RO-affected teeth is to promote bone growth.

The edentulous areas are restored with a partial removable denture to maintain masticatory function and preserve satisfactory occlusion, thereby avoiding supraposition of opposing teeth and preserving space and normal vertical dimensions. For the patient, the aesthetic function is very important in order to lessen the psychological effects of premature tooth loss or missing teeth.^24^ A partial removable denture is relatively easy and inexpensive to manufacture, compared to implants and prosthetics. Furthermore, it can be made at any age. However, a removable denture may have a negative effect on the quality of life.^27,32^

Generally, implant treatment is carried out after completed growth, e.g. cases D, E and F and in the study by Fergusson et al.^17^ Attempting to eliminate the supraposition and give the patient a temporary cosmetically and functionally acceptable treatment and increased quality of life, early implants were inserted at age 6 in patient C (Fig 1). The motivation for the very early implant insertion was to give the patient a fixed prosthetic solution from the start. As there were no teeth left in the bone to stimulate alveolar growth, the implants were inserted based on experience in patients with ectodermal dysplasia.^6,29^ The use of early implants can have a cosmetic benefit, may lessen the psychological effect of wearing a partial removable denture at a young age, and increase the quality of life. However, implants do not stimulate growth in the alveolar bone and they cannot be moved, in contrast to auto-transplanted teeth or RO-affected teeth.

Implants may also be an option when orthodontic anchorage is needed when levelling overeruption, e.g. in patients C, D, and G. A palatal temporary implant was used to level the occlusal plane in the maxilla in patients D and G. In patient D, a dental implant in the mandibular premolar region was inserted initially to promote straightening up the second molar and later used for a prosthetic solution.

In patient G, auto-transplantation was attempted, but was only partially successful, as one of the teeth ankylosed. Others have performed similar treatments with good results.^8,54^ When performing auto-transplantation, extensive orthodontic treatment is frequently required. A crucial factor for success is timing. One has to wait for the most favourable time of root formation of the donor tooth, and unfortunately there is a risk of tooth loss.

The pros and cons of auto-transplantation vs implant treatment should be individually weighed when choosing treatment. If reduced bone growth causes complications, bone augmentation, inter-positional sandwich osteotomy or bone-block repositioning may be viable solutions.

Orthodontic treatment was conducted in all our patients as part of the treatment when permanent teeth were present, although this is only described in two cases in Table 2. Orthodontic treatment is indicated both if teeth with RO are to be preserved to facilitate eruption and after extraction in some cases.

One of the major problems with RO being the cause for extraction, and thereby tooth loss, is the occurrence of pulp necrosis and dental abscesses. Abscesses are often caused by bacterial ingrowth due to the poorly mineralised enamel and dentin or dentin clefts and widened dentin tubules or invaginations.^14^ This problem is worsened by the fact that the teeth erupt so slowly that applying a composite or a temporary crown as a protective shield on the teeth is rarely possible. In three of our patients (D, F, and G), progressive deposition of hard tissue and reduction of pulp lumen were seen. However, in patient F, this development was not completed before an abscess developed. More favourable outcomes have been described by Ganguly et al,^18^ Cho^12^ and Koruyucu et al,^30^ showing that it is possible to observe maturation and thus preserve teeth with RO.

The literature describing successful endodontic treatment in RO-affected teeth is sparse.^31,52^ This concept was also attempted in one of our patients, but eventually the teeth had to be extracted due to persistent infection. Quinderé et al^43^ performed endodontic treatment of RO-affected first permanent molars and incisors, but they did not mention the type of filling and no follow-up radiographs were presented. New biocompatible materials such as MTA (mineral trioxide aggregate) or Biodentine may offer new opportunities, and have been used successfully by Koruyucu et al.^30^

Preserving RO-affected teeth can lead to a long treatment course and be substantially labor intensive, as in the case described by Koruyucu et al.^30^ At first, the teeth were temporarily prosthetically rehabilitated. Periodontal surgery followed, then orthodontic treatment, then endodontics with MTA, then fiberposts and finally zirconia crowns.

The burden of care in RO should to be addressed. It is well known from the literature that complex treatments extending over a long period of time and including several dental specialities may create an excessive ‘burden of care’ for the patient.^45^ In the present study, one of the patients (patient G) experienced a very long course of treatment. This patient had severe behavioural management problems at a young age due to multiple single tooth extractions and needed periods without treatment, which the treating dentist had to accept. One may attempt to lessen the burden of care for the child through bundling extensive treatments and performing some under general anaesthesia, even if it means removing multiple teeth at one time. Although this would eliminate the possibility of preserving some of the teeth, it may also avoid the risk of pain in the small child.

Correspondingly, an ideal treatment plan not only considers the length of treatment course, but also the need to minimise the burden of care to avoid treatment fatigue. In the present study, this treatment approach was best exemplified by patient C, in whom all treatment of permanent affected teeth and insertion of dental implants were performed under general anaesthesia in three consecutive sessions at the age of 6 years, as the patient did not accept regular dental visits. The patient will later need correction, orthodontic treatment and possibly orthognathic surgery. Hopefully, the patient may gain more understanding and acceptance of the treatment as s/he matures.

In order to plan optimal treatment, early referral of patients with RO is paramount. In our oldest patients, 2-3 years passed from diagnosis until referral to RCROD, while the youngest (most recent) patients experienced only a short time between diagnosis and referral to RCROD. It is this group’s experience and opinion that establishment of specialised, interdisciplinary teams such as the RCROD’s will increase knowledge and awareness among dentists about rare dental diseases like RO. It is hoped that this will optimise treatment by increasing the quality of dental rehabilitation as well as decreasing the burden of care for patients in the future.

## Conclusion

RO is a localised hypomineralisation condition with unknown origin affecting the primary and permanent dentition. The case reports in Table 1 and 2 show RO occurring most frequently in the maxillar. Our cases showed higher frequency of RO in males, contrary to previous findings, but RO presents in both sexes. The choice and timing of treatment is extremely challenging for paediatric dentists and one must carefully weigh the pros and cons of a conservative or more radical treatment plan.

The treatment of RO is typically extensive and begins at an early age, which is why these patients suffer from a high burden of care.

Knowledge about the condition and treatment in specialised teams would improve the treatment and reduce the patients’ burden of care.
